# Supplementary material for: Comprehensive Annotation and Functional Exploration of MicroRNAs in Lettuce
Source: Front Plant Sci. 2021 Dec 24;12:781836. doi: 10.3389/fpls.2021.781836 (PMC8739914; doi:10.3389/fpls.2021.781836)
Supplement: Supplementary file 3 [file Data_Sheet_9.ZIP › GSM2230751.plot/Lsa-miRN35_Lsat_1_v5_gn_6_34600.1_1882_TPlot.pdf]

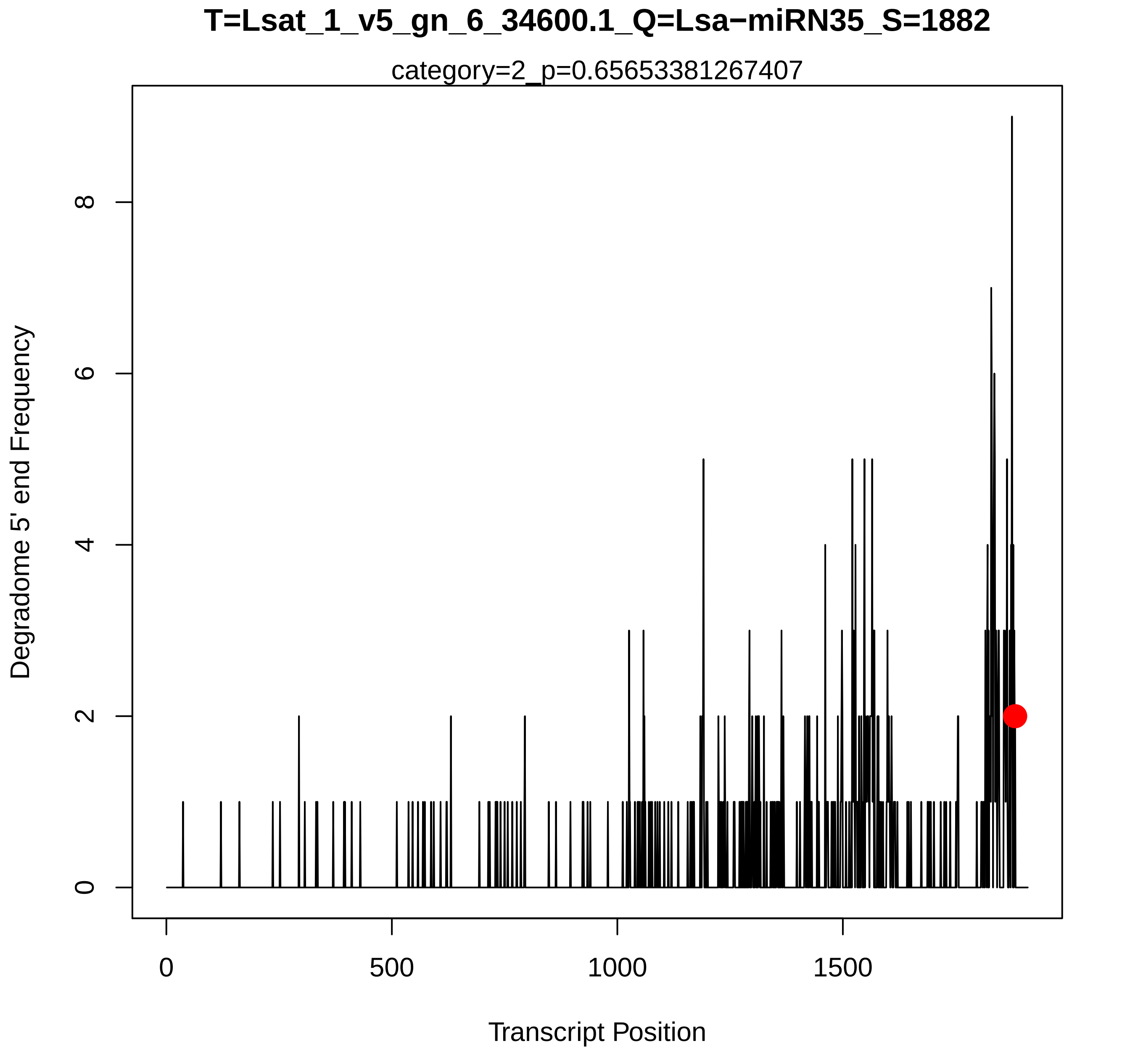

T=Lsat_1_v5_gn_6_34600.1_Q=Lsa−miRN35_S=1882
category=2_p=0.65653381267407
Degradome 5' end Frequency
0
2
4
6
8
0
500
1000
1500
Transcript Position
